# Intraneural Hemangioma: A Report of Two Cases

**DOI:** 10.7759/cureus.105778

**Published:** 2026-03-24

**Authors:** Jodie B Thomas, Shreyas Panchagnula, Wendy S Towers, Nima Sharifai, Khalid H Kurtom

**Affiliations:** 1 Neurosurgery, University of Maryland Shore Regional Health, Easton, USA; 2 Neurosurgery, University of Maryland Medical Center, Baltimore, USA; 3 Pathology, University of Maryland School of Medicine, Baltimore, USA

**Keywords:** benign spindle cell proliferation hemangioma, intraneural hemangioma, minimally invasive spine surgery, nerve sheath hemangioma, spinal hemangioma

## Abstract

Spinal hemangiomas are congenital vascular malformations. Most reports of spinal hemangiomas describe resection via traditional open approaches; however, advances in minimally invasive surgery have led to comparable results. We report on two cases, namely, one nerve root intraneural hemangioma and one nerve root extraforaminal hemangioma, both removed utilizing a minimally invasive technique.

Case 1 is about a 61-year-old woman who presented with low back pain with left lower extremity radiculopathy refractory to maximum medical management. This was secondary to an enhancing mass at the right L4-L5 neural foramen (2.4×2.2×2.1 cm) with impingement of the right L4 nerve root. A minimally invasive right L4 nerve sheath mass resection with right-sided L4-L5 transforaminal interbody fusion and pedicle screw fixation was performed. Final pathology was consistent with a benign spindle cell proliferation, indicative of hemangioma.

Case 2 is about a 52-year-old man who presented with low back pain with bilateral lower extremity radiculopathy. This was secondary to an enhancing intradural extramedullary lesion (1.4×0.8×1.1 cm) in the spinal canal at the L4 vertebral body with resulting canal stenosis. A minimally invasive L4 intradural spinal mass resection was performed. Final pathology was consistent with intraneural hemangioma.

## Introduction

Spinal hemangiomas are congenital vascular malformations whose pathologies are considered to be local malformations made up of an abnormal mixture of cells and tissue [1,2]. Spinal epidural hemangiomas (SEHs) can be divided into two types: epidural hemangiomas of vertebral origin and primary epidural space hemangiomas [1,3-5]. SEHs account for 4% of all spinal epidural tumors, mostly occurring as primary lesions in the vertebral bone [1,6]. Historically, resection of these lesions via traditional open approaches can create morbidity that can rival the tumor's symptoms. Advances in minimally invasive surgery (MIS) have led to comparable results with other intradural pathologies [7-10]. This shift from exposure-based surgery to precision-based surgery allows for smaller incisions, less muscle destruction, reduced blood loss, and a shortened hospital stay.

We report two cases of spinal hemangiomas: one nerve root intraneural hemangioma and one nerve root extraforaminal hemangioma. Both cases were resected using the MIS approach through a tubular retractor system. These cases demonstrate advancement in minimally invasive techniques for the surgical management of spinal hemangiomas.

## Case presentation

Case 1

Initial Presentation

**Figure 1:**
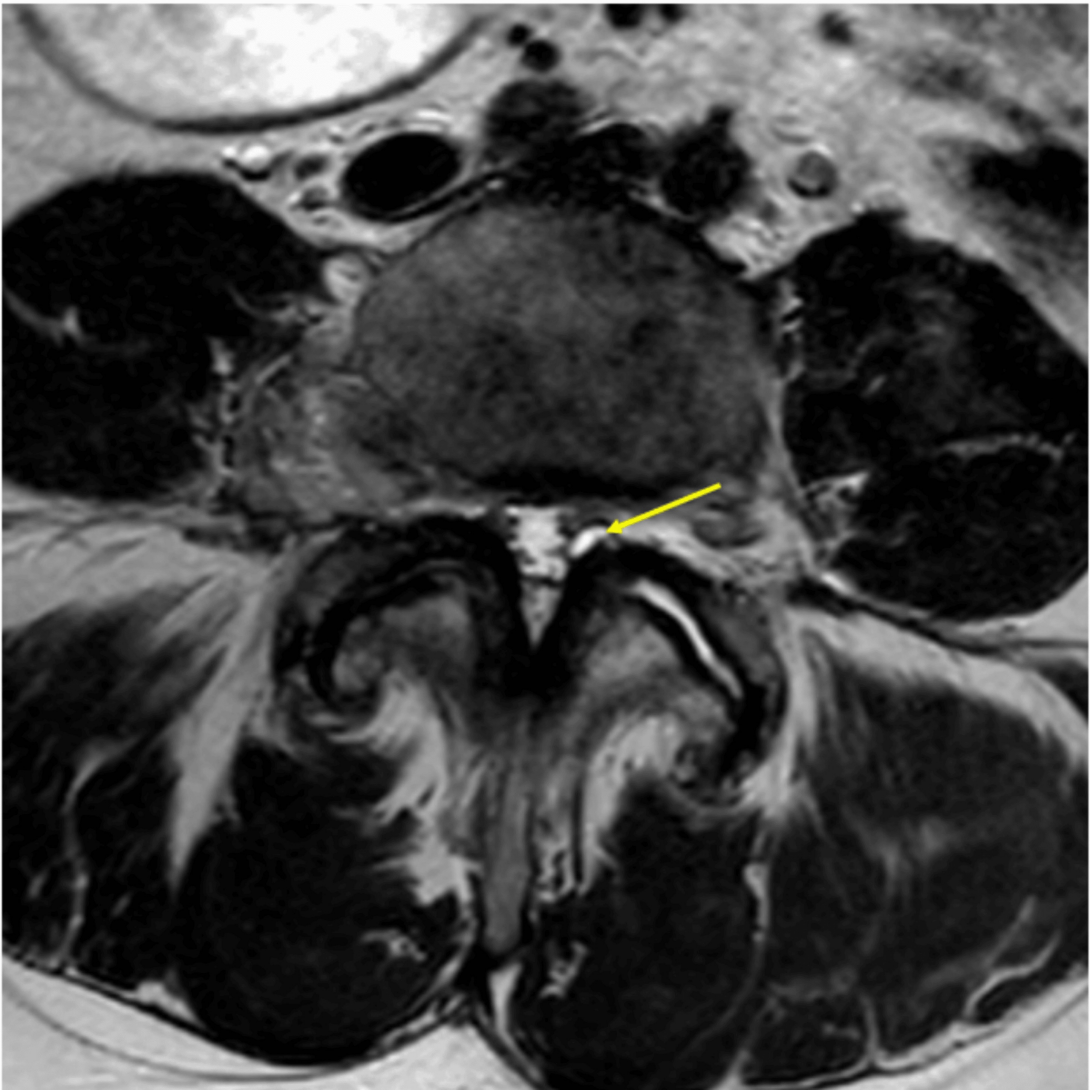
Preoperative T2 axial image demonstrating a left synovial cyst

**Figure 2:**
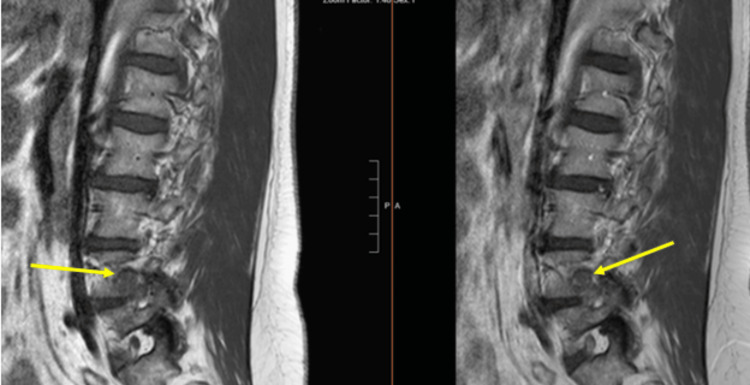
Preoperative T1 sagittal image (a) without and (b) with gadolinium demonstrating an enhancing mass at the right L4-L5 neural foramen (2.4×2.2×2.1 cm) with impingement of the right L4 nerve root

**Figure 3:**
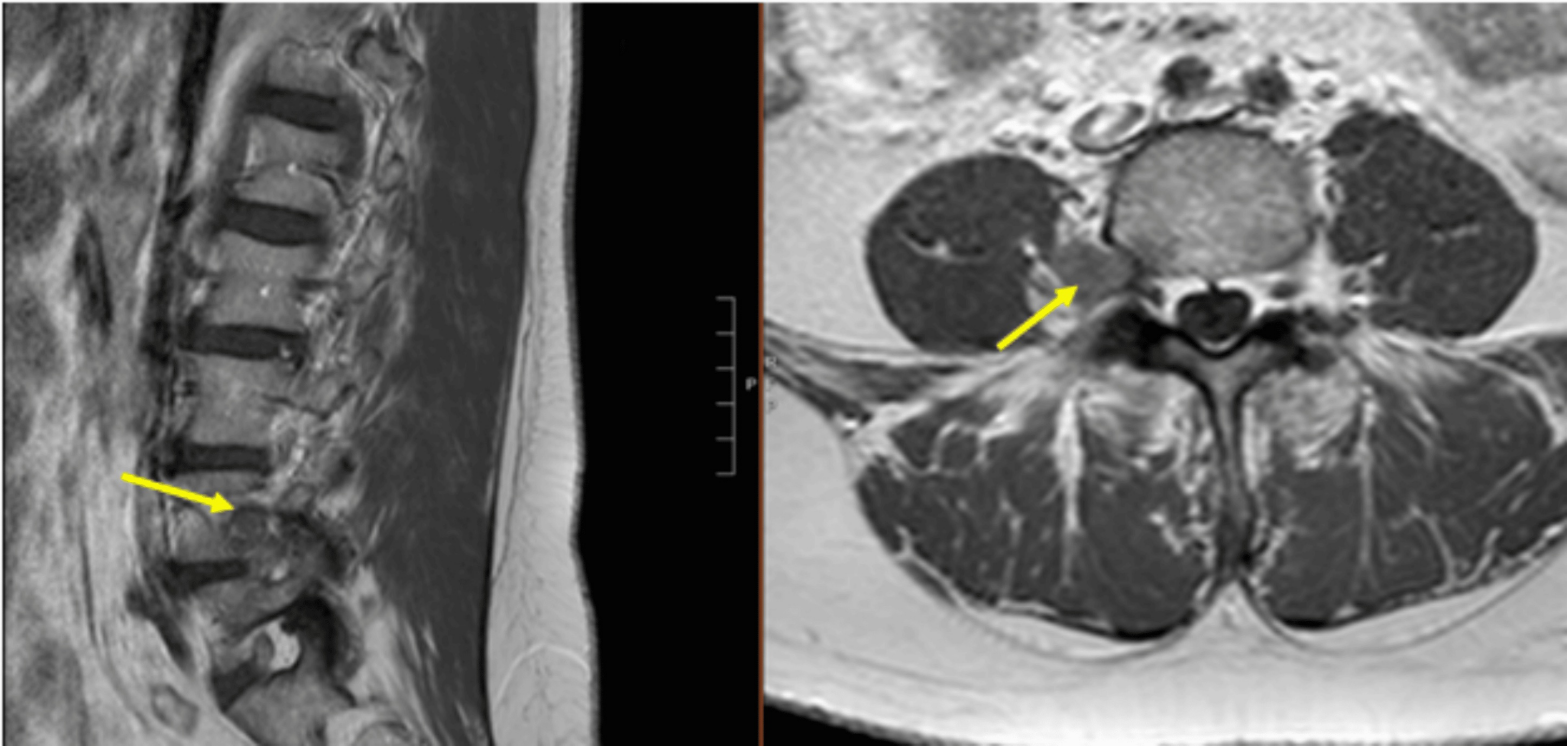
Preoperative (a) sagittal and (b) axial image with gadolinium

**Figure 4:**
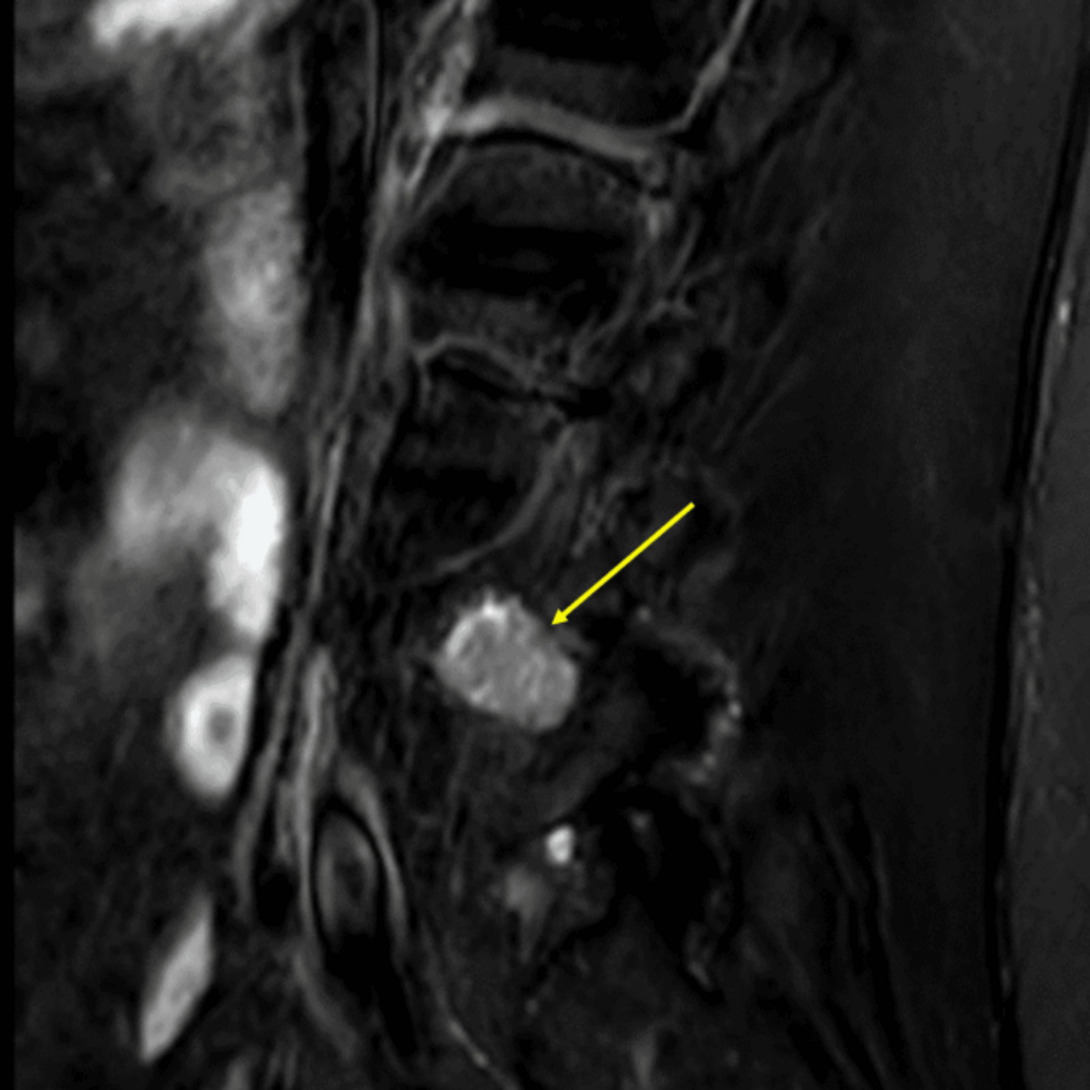
Preoperative STIR sequence STIR: short tau inversion recovery

A 61-year-old woman presented to the clinic with low back pain radiating down the left lower extremity, with significant impact on daily living and minimal improvement after conservative management, including epidural injections and physical therapy. Clinical examination revealed bilateral quadriceps and anterior tibialis weakness demonstrating 4/5 power. Sensory deficits were also present in the L4 and L5 distribution bilaterally, suggestive of radiculopathy. Bilateral patellar reflexes were depressed. Magnetic resonance imaging (MRI) demonstrated evidence of degenerative disease, particularly at the L4-L5 level with central stenosis secondary to annular bulge, facet arthropathy, and ligamentum flavum buckling. Notably, protruding from the left facet is a small synovial cyst with consequent neuroforaminal narrowing and exiting L4 nerve impingement (Figure 1). Contrast MRI revealed an enhancing mass at the right L4-L5 neural foramen (2.4×2.2×2.1 cm) with impingement of the right L4 nerve root (Figures 2-4). Smooth remodeling of the posterior cortex of L4 and widening of the neural foramen were suggestive of a long-term, indolent process from the development of a benign neoplasm such as a nerve sheath tumor. High on the differential diagnosis was schwannoma or neurofibroma. The patient underwent a minimally invasive right L4 nerve sheath mass resection with right-sided L4-L5 transforaminal interbody fusion and pedicle screw fixation. Of note, through this approach, the contralateral symptomatic degenerative pathology was also addressed. The contralateral synovial cyst resection as well as direct foraminal decompression was achieved. Histologic analysis of the nerve sheath tumor demonstrated segments of fibrous tissue, adipose tissue, and bone fragments with foci of bland spindle cell proliferation surrounding dilated capillaries, indicative of hemangioma.

Surgical Technique

**Figure 5:**
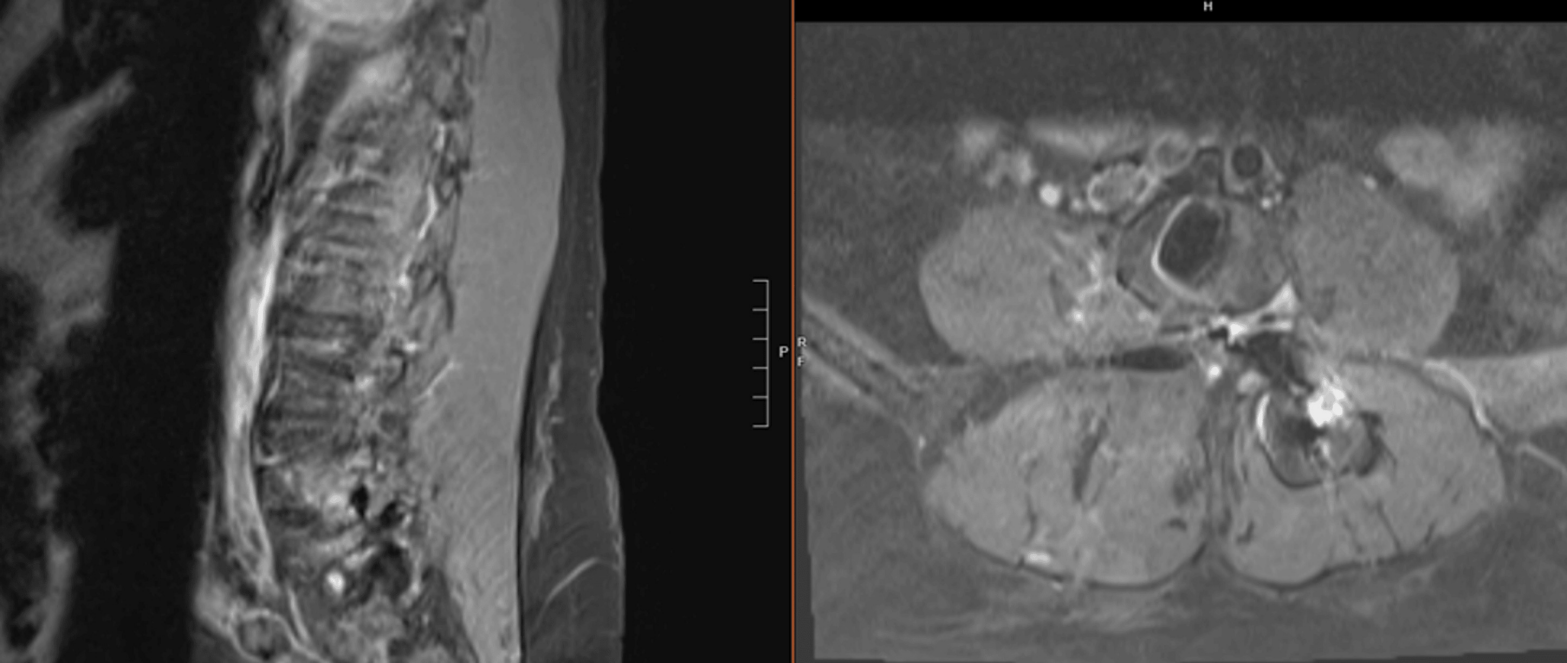
Postoperative T1 (a) sagittal and (b) axial image with gadolinium demonstrating the gross total resection of the lesion

**Figure 6:**
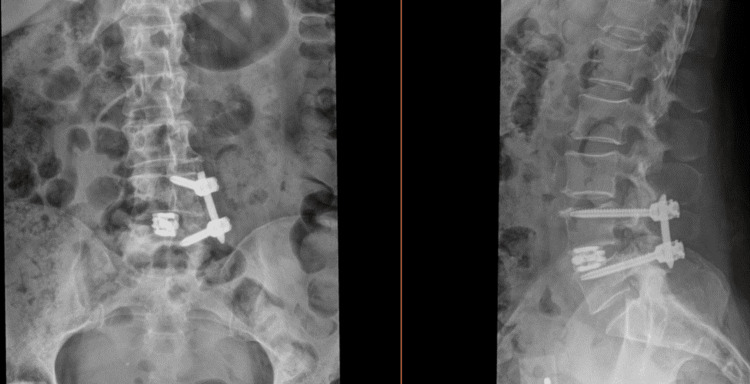
Postoperative (a) posterior and (b) lateral X-ray of left L4-5 pedicle screw fixation with interbody titanium graft

The patient was placed prone on a Jackson table with slip pads, and a fluoroscopy machine was then brought in to localize the area of interest at L4-L5. An official timeout was taken to confirm the patient's identification, the availability of preoperative imaging, as well as the correct location of surgery. The patient was then prepped and draped in standard surgical fashion. The 30 mL of 0.25% Marcaine with epinephrine was used for local skin analgesia. Two small 1-inch incisions were made approximately 4 cm off the midline over the patient's L4-L5 region. Dissection was made down to the fascia, which was then sharply cut. Jamshidi needles followed by K-wires were then inserted transpedicularly into the left L4 and L5 pedicles. This was done with anteroposterior (AP) and lateral fluoroscopy imaging as well as continuous electromyography (EMG) neuromonitoring. The minimally invasive tubular retractor system was then placed over the patient's right L4-L5 facet. This was all confirmed with fluoroscopy imaging. The electric drill, various curettes, Kerrison rongeurs, and pituitaries were then used to complete a total facetectomy at the right L4-L5 facet, bilateral decompressive laminectomies through a laminoplasty approach, resection of the ligamentum flavum, as well as bilateral foraminotomies. There was a large synovial cyst on the patient's left L4-L5 region that was removed completely, achieving complete decompression of the thecal sac as well as bilateral neural foramina. This also allowed access to the disk space laterally. A complete discectomy was then achieved using various pituitaries and curettes. A copious amount of antibiotic irrigation was used throughout the disk space. Bipolar electrocautery and FloSeal (Baxter, Deerfield, Illinois, United States) were used to establish hemostasis. The interbody and the posterolateral fusion were then completed by packing morselized autograft and allograft material into the disk space as well as in the posterolateral margin to achieve a posterolateral fusion. The same material was also packed within the interbody cage. The cage was placed with AP and lateral fluoroscopy imaging. Attention was then paid to the right L4 nerve root. We followed the nerve root laterally as it entered the psoas muscle. There was a large beefy hemorrhagic loosely adherent mass that was compressing the nerve root and was attached to it. This mass did not appear to be entering the nerve sheath; however, it was attached to the outside of the sheath presumably coming from the sheath itself. This lesion was removed using various pituitaries, curettes, and microinstruments with the aid of the intraoperative microscope. The nerve root was completely decompressed from the spinal canal all the way out laterally within the iliopsoas muscle, achieving gross total resection of the lesion (Figure 5). Bipolar electrocautery and FloSeal were used to establish hemostasis. The tubular retractor system was then removed, and the surgery was completed by placing percutaneous screws over the K-wires on the left side at L4-L5. They were connected with a titanium rod in a percutaneous minimally invasive fashion, achieving complete fixation in the segment. To ensure that serial imaging postoperatively can be completed without artifact, it was determined that unilateral fixation of the contralateral side was appropriate (Figure 6). There were no complications, and blood loss was minimal. The patient was discharged home the same day moving all extremities with good strength and ambulatory in the post-anesthesia recovery unit. Preliminary pathology in this case was consistent with a nerve sheath tumor.

Pathology

**Figure 7:**
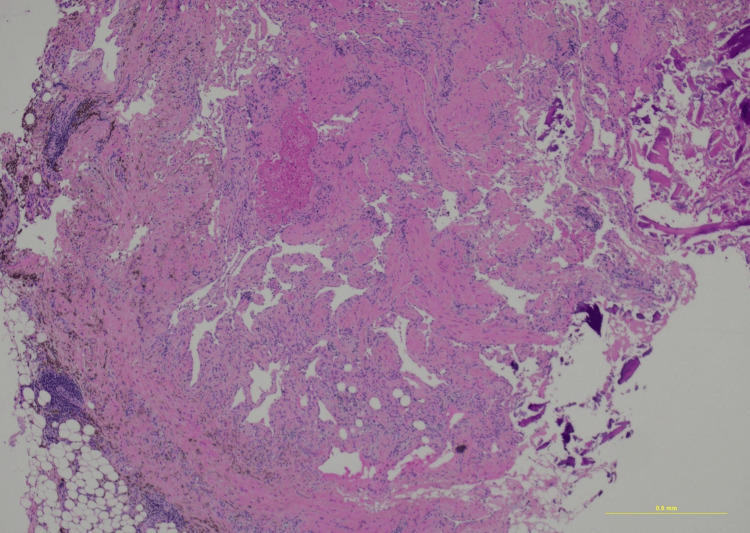
Hematoxylin and eosin staining, 4× power

**Figure 8:**
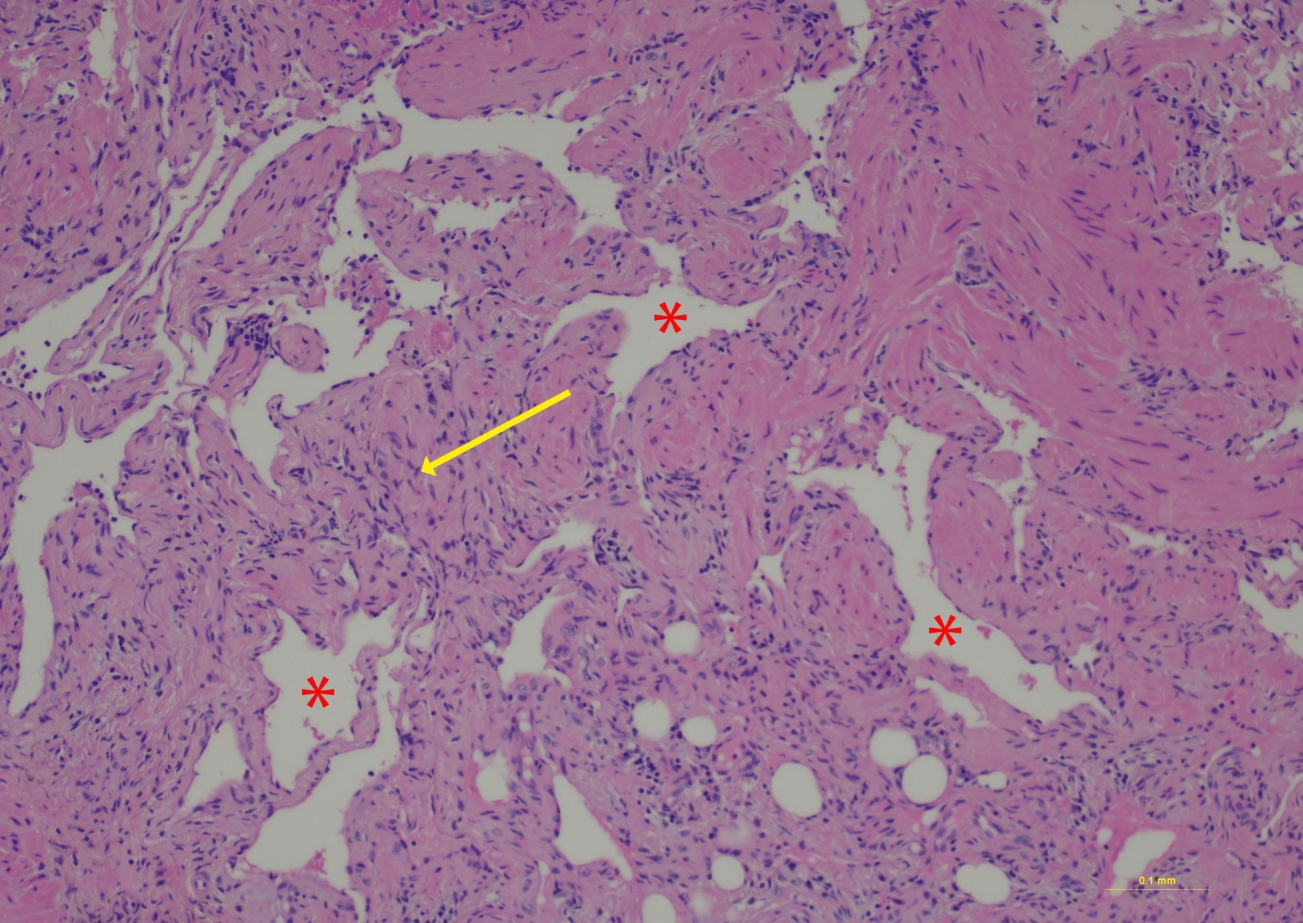
Hematoxylin and eosin staining, 10× power. Red asterisks highlight the slit-like vascular spaces. Yellow arrow indicates the spindle cell (fibroblast) component present within the intervening fibrous areas

A fibrovascular lesion demonstrated peripheral hemosiderin deposition (Figures 7-8), consistent with prior hemorrhage.** **The lesion consists of a bland spindle cell proliferation and dilated vascular spaces. Final diagnosis was consistent with a benign spindle cell proliferation, indicative of hemangioma.

Postoperative Course and Follow-Up

The patient's back pain progressively improved postoperatively at one month with continued paresthesias extending to her feet bilaterally. She was pain-free at her three-month postoperative evaluation. Serial imaging has shown no evidence of recurrence thus far.

Case 2

Initial Presentation

**Figure 9:**
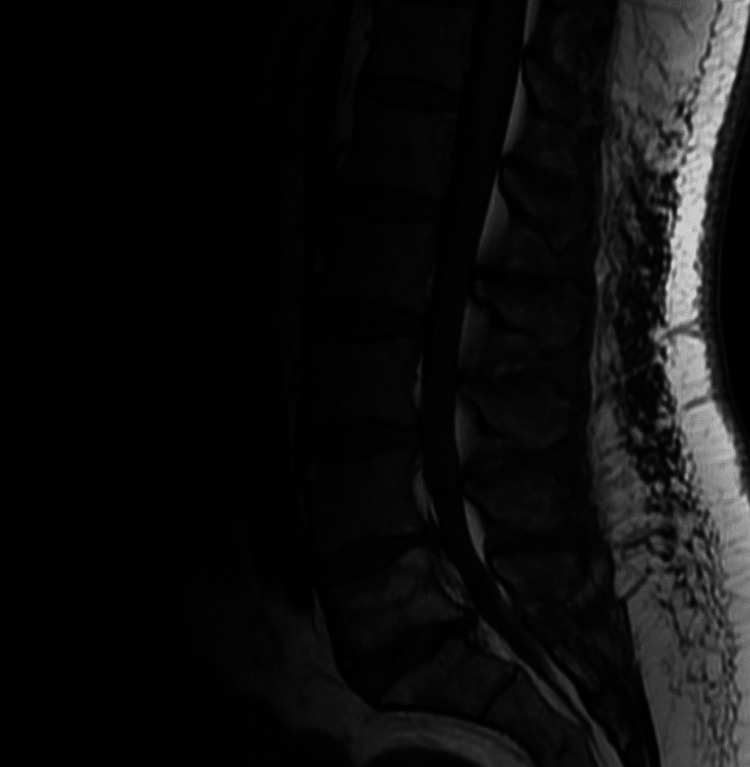
Preoperative T1 sagittal image without gadolinium

**Figure 10:**
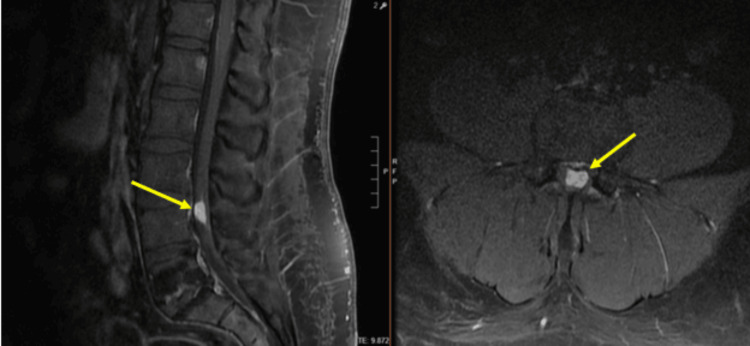
Preoperative T1 (a) sagittal and (b) axial image with gadolinium demonstrating an enhancing intradural extramedullary lesion (1.4×0.8×1.1 cm) in the spinal canal at the L4 vertebral body with resulting canal stenosis

**Figure 11:**
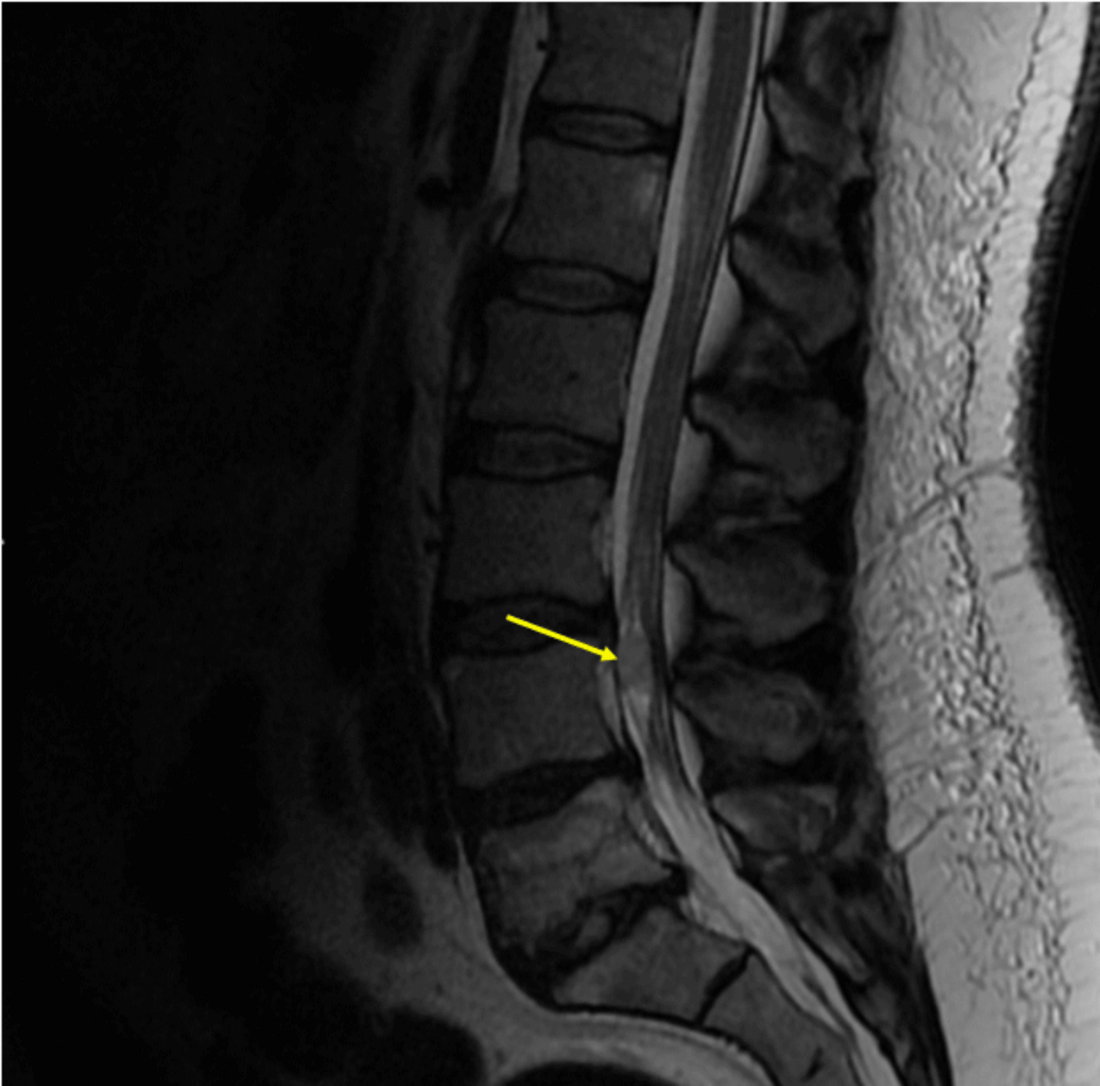
Preoperative T2 sagittal image

A 52-year-old man presented to the clinic with a history of a motor vehicle collision several months prior with low back pain radiating to bilateral lower extremities. He had associated intermittent numbness of his bilateral lower extremities which was worse in the supine position. There was no weakness noted on the exam. With his continued symptoms despite conservative management, an MRI was ordered which discovered a spinal mass, and the patient was referred to neurosurgery. Lumbar spine MRI with and without gadolinium demonstrated an enhancing intradural extramedullary lesion (1.4×0.8×1.1 cm) in the spinal canal at the L4 vertebral body with resulting canal stenosis (Figures 9-11). High on the differential diagnosis was schwannoma or neurofibroma. The patient underwent minimally invasive L4 intradural spinal mass resection. Pathologic analysis revealed the mass to be an intraneural hemangioma with lesional cells positive for ERG and CD3 along with an extensive reticulin network surrounding vessels in the lesion.

Operative Technique

**Figure 12:**
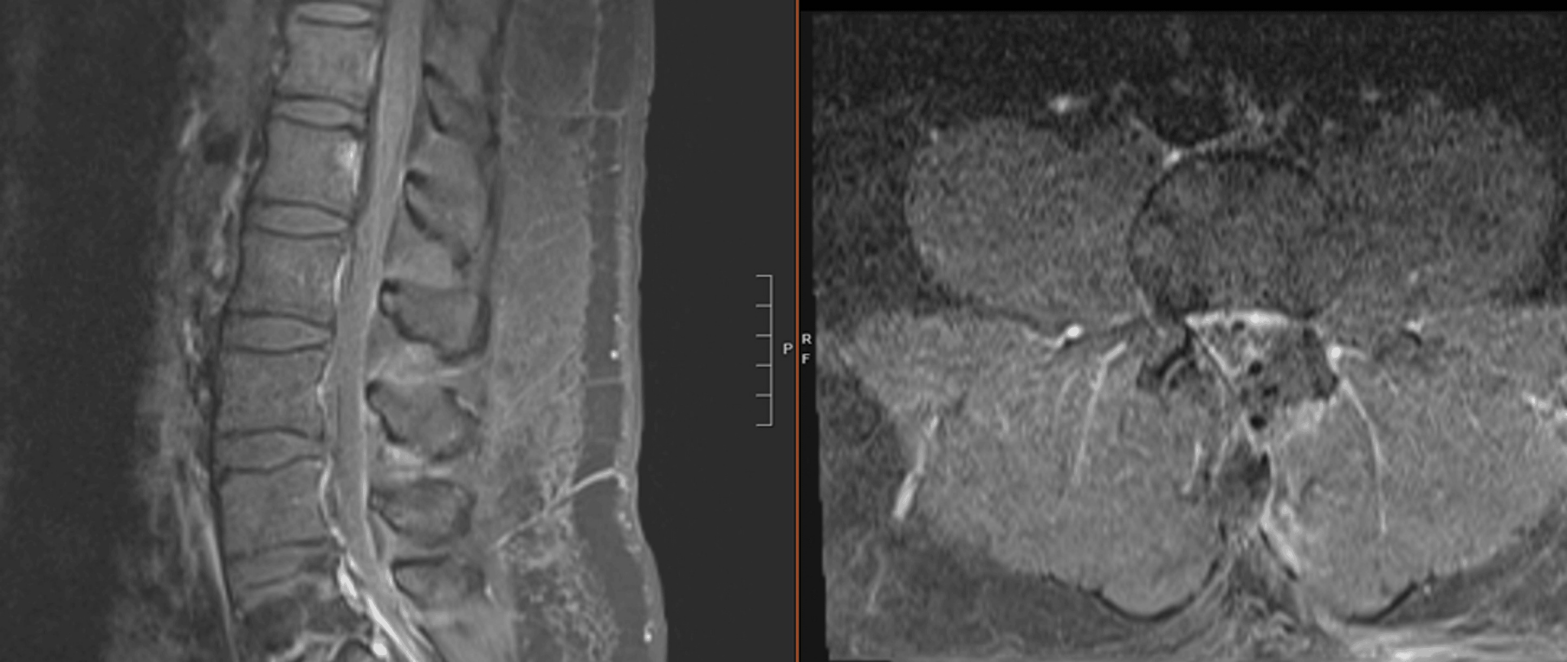
Postoperative T1 (a) sagittal and (b) axial image with gadolinium demonstrating the gross total resection of the intradural lesion

The patient was placed prone on a Jackson table with slip pads, and a fluoroscopy machine was then brought in to localize the area of interest at L4. An official timeout was taken to confirm the patient's identification, the availability of preoperative imaging, the procedure to be performed, as well as the correct location of surgery. The patient was then prepped and draped in standard surgical fashion. After 30 mL of 0.25% Marcaine with epinephrine was used for local skin analgesia, a small 1-inch incision was made approximately 1.5 cm off the midline over the patient's left L4 vertebral body. Dissection was made down to the fascia, which was then sharply cut. The minimally invasive tubular retractor system was then placed over the patient's L4 vertebra posteriorly. This was confirmed with AP and lateral fluoroscopy imaging for a second timeout. The electric drill, various curettes, Kerrison rongeurs, and pituitaries were used to complete a decompressive laminectomy at L4 with bilateral foraminotomies through a laminoplasty approach. Once exposure was achieved, the intraoperative microscope was brought in to complete the resection. The dura was then opened slightly off the midline over the patient's left side and tacked up to allow adequate exposure of the intradural space. The arachnoid was then opened and tacked up with clips. At this point, there was a large lesion seen filling the entire spinal canal, compressing the nerve roots. Careful microdissection was undertaken to isolate this mass from the surrounding nerve roots. It was clear that it was stemming from a single nerve root both proximally and distally. The nerve root was isolated with careful microdissection. Intraoperative neuromonitoring was then conducted by stimulating the nerve root proximally and distally without any stimulation at 1 milliamp. The nerve root was then coagulated proximally and distally and cut. The mass was removed en bloc with the attached cut nerve root (Figure 12). Closure was completed using microvascular clips for the dura which allows for tight dural closure in a small tubular MIS field. This was followed by a piece of TachoSil (Corza Medical, Boulder, Colorado, United States) with the application of Tisseel (Baxter, Deerfield, Illinois, United States) to achieve a watertight closure. Multiple Valsalva maneuvers were then applied without any evidence of cerebrospinal fluid (CSF) leak. Closure was then completed in standard fashion. There were no complications, and blood loss was minimal. The patient was extubated and taken to the post-anesthesia care unit in good condition, moving bilateral lower extremities with good strength. Somatosensory evoked potential (SSEP) and motor evoked potential neuromonitoring remained stable throughout the case. Preliminary pathology in this case was consistent with a vascular lesion.

Pathology

**Figure 13:**
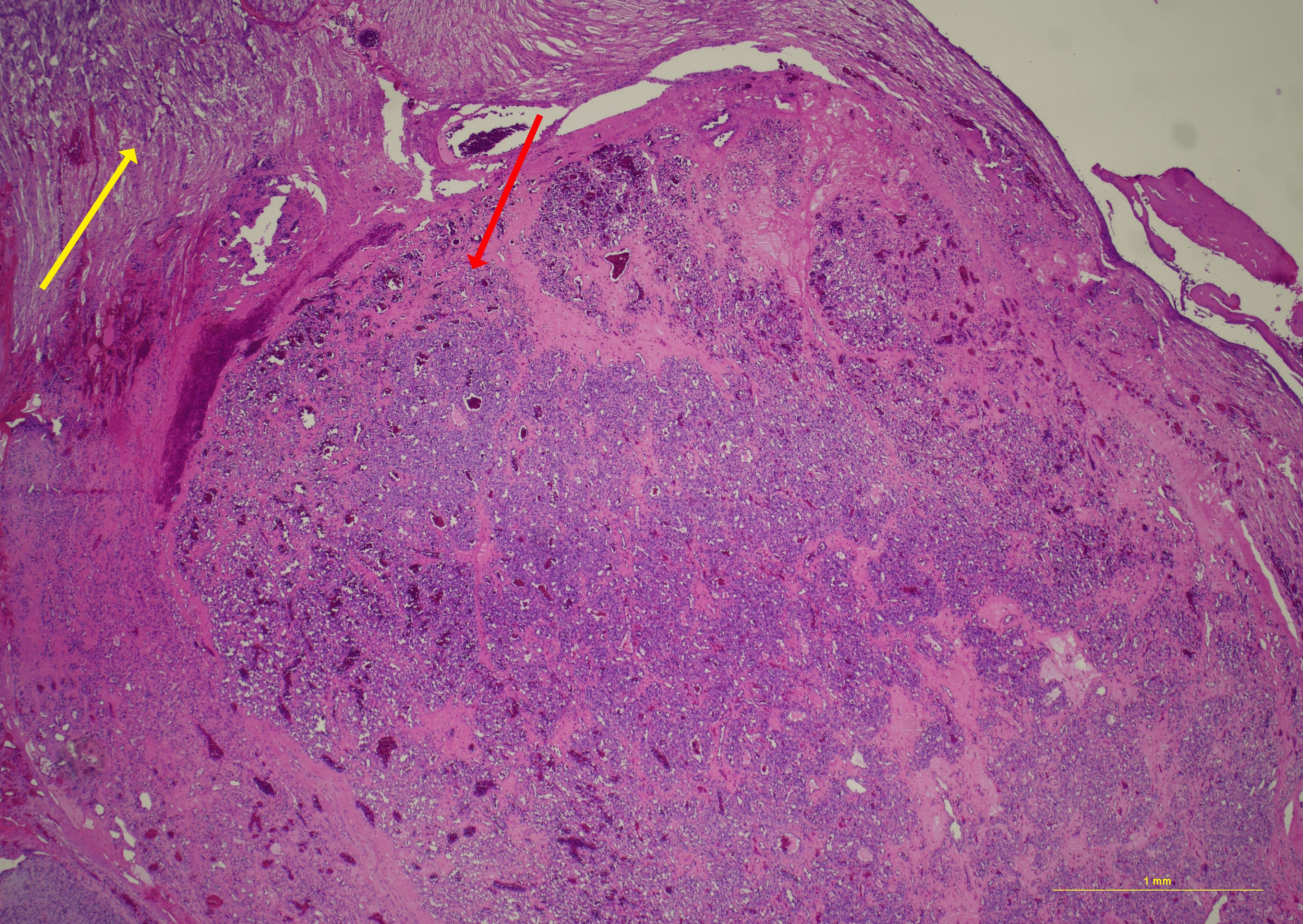
Hematoxylin and eosin staining, 2× power. The nerve (yellow arrow) is seen to contain an intraneural hemangioma (red arrow), with an abrupt transition at the point of involvement

**Figure 14:**
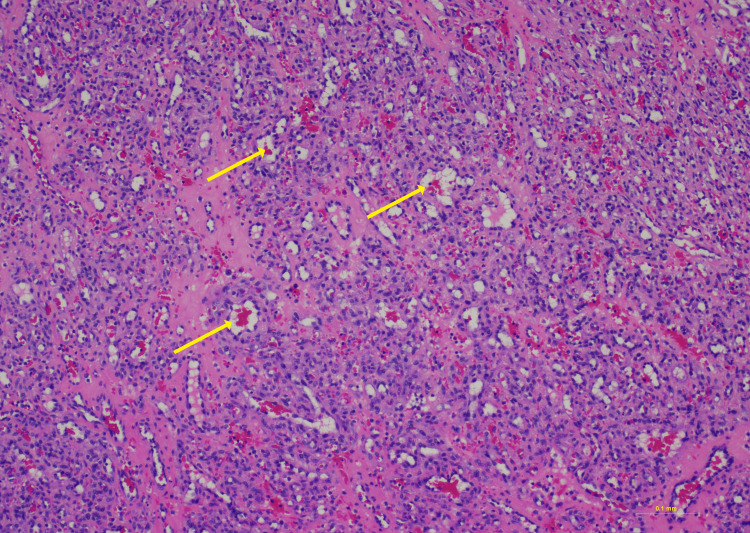
Hematoxylin and eosin staining, 10× power. Numerous small capillary lumens (yellow arrows) comprise the hemangioma

In Figure 13, the nerve fascicle can be seen (top left) transitioning into a well-circumscribed hemangioma (bottom right). The hemangioma consists of a proliferation of tightly packed capillaries with intervening stroma (Figure 14). The endothelial cells do not demonstrate significant cytologic atypia or mitotic activity, as expected for this benign lesion. Final diagnosis was consistent with intraneural hemangioma.

Postoperative Course and Follow-Up

**Figure 15:**
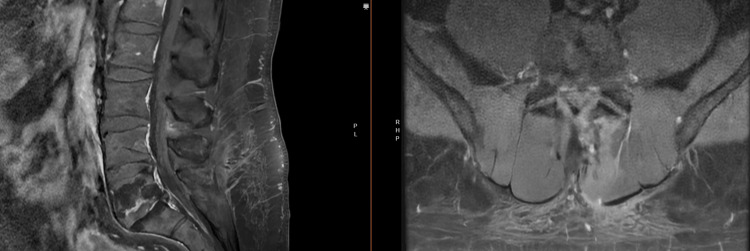
Eight months post-op T1 (a) sagittal and (b) axial image with gadolinium

Bilateral lower extremity paresthesia resolved on postoperative day 1; however, he did develop transient weakness of the left lower extremity. At the initial two-week postoperative visit, no paresthesias were noted, and the weakness had resolved.** **MRI at the six-month follow-up did not show recurrence of the lesion (Figure 15).

## Discussion

Primary tumors of the cauda equina comprise only about 6% of spinal cord tumors [11,12]. About 2-7% of all intraspinal tumors are of vascular origin [11-13]. We report two cases of spinal hemangiomas resected through a tubular MIS technique: one nerve root intraneural hemangioma and one nerve root extraforaminal hemangioma in the lumbar spine. Hemangiomas, either capillary or cavernous, have been rarely encountered in the spinal intradural space [14,15]. Because of its embryological origin, the most common tumor site is the dorsal or dorsolateral spinal canal where the venous plexus is abundant [1,16]. Nowak et al. reported a case of capillary hemangioma growing within the boundaries of the nerve trunk of the cauda equina [11]. Previous reports of capillary hemangiomas mainly described intraneural hemangiomas of the peripheral nerves [17]. Differential diagnoses for enhancing intradural tumors are schwannoma, meningioma, hemangioblastoma, hemangioma, paraganglioma, drop metastasis, lymphoma, and ependymoma [14]. The most common intradural tumors are schwannoma and meningioma [14]. In both of these cases, high on the differential diagnosis was schwannoma or neurofibroma. However, in retrospect, the marked hyperintense T2 signal and the intense enhancement in Figure 10 suggest the flow into the vascular channels of the lesion [18,19]. The short tau inversion recovery (STIR) sequence noted in Figure 4 also portends that the lesion in the first case may be fluid-filled, suggestive of a vascular structure [18,19]. As illustrated in Sanghvi et al.'s case, sinusoidal vascular structure should be considered in the differential diagnoses of spinal epidural soft tissue masses [19].

Traditional open approaches, though allowing for wider exposure of the operative field, require greater dissection of the paravertebral muscle with laminectomy and, oftentimes, facetectomy, requiring the use of fusion to ensure stability [20]. MIS approaches have been shown to effectively treat thoracolumbar nerve sheath tumors, achieving gross total resection of the tumor along with reduction in blood loss, intraoperative and postoperative pain medication, shorter inpatient stay, faster return to work, and overall cost-effectiveness for healthcare systems [21].

As this case report is limited to two procedures, we cannot offer comprehensive recommendations to utilize the MIS approach for all nerve sheath tumor resections. However, we encourage the publication of MIS approaches for nerve sheath tumor resections in order to further expand MIS capability to spinal tumor resection. 

## Conclusions

Intraneural and extraforaminal hemangiomas are very rare spinal tumors. Advances in MIS have allowed for the resection of intradural pathologies with less tissue damage, minimal blood loss, and a shortened recovery period. As illustrated in this report, MIS techniques are an effective means for the resection of spinal hemangiomas, and although rare, this method should not be excluded as a means of surgical care. These cases add to the growing literature that supports MIS techniques as safe and effective when treating nerve sheath tumors. Larger case series and randomized controlled trials are warranted in order to establish MIS approaches as the standard of care in the neurosurgical treatment of spinal hemangiomas and other nerve sheath tumors.
